# Outlook of pandemic preparedness in a post-COVID-19 world

**DOI:** 10.1038/s41541-023-00773-0

**Published:** 2023-11-20

**Authors:** B. Adam Williams, Charles H. Jones, Verna Welch, Jane M. True

**Affiliations:** Pfizer, 66 Hudson Boulevard East, New York, NY 10001 USA

**Keywords:** Viral infection, Public health

## Abstract

The COVID-19 pandemic was met with rapid, unprecedented global collaboration and action. Even still, the public health, societal, and economic impact may be felt for years to come. The risk of another pandemic occurring in the next few decades is ever-present and potentially increasing due to trends such as urbanization and climate change. While it is difficult to predict the next pandemic pathogen threat, making reasonable assumptions today and evaluating prior efforts to plan for and respond to disease outbreaks and pandemics may enable a more proactive, effective response in the future. Lessons from the COVID-19 response and pandemic influenza preparedness underscore the importance of strengthening surveillance systems, investing in early-stage research on pandemic pathogens and development of platform technologies, and diversifying response plans across a range of tactics to enable earlier access to safe and effective interventions in the next pandemic. Further, sustaining the robust vaccine manufacturing capacity built because of COVID-19 will keep it ready for rapid response in the future. These actions will not be successful without improved global coordination and collaboration. Everyone, including the biopharmaceutical industry, has a role to play in pandemic preparedness, and working together will ensure that the most lives are saved in the next pandemic.

## Introduction

The substantial public health and societal costs of the COVID-19 pandemic are wide-ranging and have been observed across the world. As of August 2023, COVID-19 has infected over 770 million people and resulted in over 6.9 million reported deaths globally^1^, although the World Health Organization (WHO) estimates mortality could be much higher based on global excess mortality^2^. It has contributed to a reduction in global life expectancy; in the United States alone, life expectancy fell by an estimated 1.8 years, with a disproportionate drop among racial minorities. Many of those infected with COVID-19 still suffer from long-term COVID, with symptoms remaining months after the initial illness^3^.

More broadly, COVID-19 led to substantial indirect health effects. Mental health declined worldwide, with a 25% increase in the global prevalence of anxiety and depression in the first year of the pandemic^4^. Disruptions to routine immunization programs in at least 68 countries affected more than 80 million children worldwide, likely resulting in higher rates of vaccine-preventable diseases^5,6^. Delays and avoidance of medical care contributed to more severe observed health outcomes; for example, delayed cancer screening and treatment have resulted in increased cancer mortality rates^7^. Beyond direct and indirect health effects, the pandemic challenged conventions around how people learn, work, and play. For example, delivering quality education virtually posed a challenge in some parts of the world, while in other parts of the world, formal education stopped altogether, setting back learning and development for an entire generation of children^8–10^. The negative health and societal impacts of COVID-19 may be felt for years to come. Unfortunately, many of these statistics and estimates will likely increase in magnitude with post-pandemic analyses, and as of the date of this publication, the entirety and extent of the pandemic’s impact are still not known.

The resulting global economic impacts of the COVID-19 pandemic were also unprecedented and may continue for years. Across countries, gross domestic product (GDP) fell by 2–4% in 2020, and the US had the worst contraction in national GDP since World War II^11,12^. This decline pales in comparison to the global economic losses extending through to 2024 which are estimated to be $13.8 trillion^13^. The effect on productivity and livelihood left governments around the world juggling priorities to quickly implement response initiatives.

Despite major economic shocks, the economic burden of the pandemic may have been worse if not for at least some prior spending on pandemic preparedness and response tactics. For example, the US National Institutes of Health (NIH) spent $17.2 billion in vaccine technology research—more than $500 million toward mRNA, virus-like particle, and nanoparticle vaccines—before 2020 with specific attention to diseases with pandemic potential^14^. These early initiatives set some of the foundational work on which new COVID-19 vaccine candidates were based. However, even with these investments, a report by a WHO-established panel found that on the whole, investment in pandemic preparedness before COVID-19 was inadequate, prompting efforts to revisit how the world prepares for the next pandemic, an ongoing risk^15^.

## The risk of another pandemic

Smaller-scale outbreaks or large-scale pandemics related to emerging infectious diseases have increased over the past century, and are projected to do so over time^16^. Evidence suggests the probability of another pandemic occurring within one’s lifetime is roughly 17% and may even grow to 44% within the next couple decades^17^. This means that in any given year, the chance of another pandemic occurring is over 2%^17^. Yet, it is difficult to determine exactly what pathogen will cause the next pandemic.

Most emerging infectious diseases with pandemic potential are initially transmitted from animals to humans—zoonotic spillover—and those that have or evolve the ability to move from human to human have the potential to become dangerous^18^. In fact, zoonotic spillover has likely been the trigger for most of the viral pandemics in the 20th century^19^. Meanwhile, societal trends are increasing the likelihood of zoonotic spillover. Urbanization and habitat destruction are placing humans and animals in closer contact, giving pathogens more opportunities to migrate to humans^20^. Climate change is also altering animal habitats and forcing them to migrate to new territories, causing many species to meet for the first time^20^. Pathogens may travel between several animal species before reaching humans; therefore, novel species interactions driven by climate change also increase the risk of zoonotic spillover^20^.

Under the right circumstances, a newly emerged pathogen can grow from a disease outbreak to pandemic proportions. The balance of a pathogen’s characteristics, including transmissibility, case fatality rate, replication rate, and mutability—among other factors—will influence the likelihood of becoming a widespread pandemic^21^. For example, pathogens with a higher case fatality rate and lower transmissibility, combined with pronounced symptomology (e.g., Ebola), are more likely to be more limited in geographic footprint. Meanwhile, SARS-CoV-2 had comparably moderate case fatality, and higher transmissibility through airborne transmission and asymptomatic spread, which led to an exponential growth in the number of infections. Additionally, societal trends that increase connectiveness between countries, like globalization and travel, can further accelerate the spread of high-risk pathogens^20^. Lastly, high pathogen mutation rates—as seen in particular with RNA viruses—also contribute to a greater risk because random mutations are a source of potential adaptations that benefit the virus. Altogether, given their high mutation rates and transmissibility, evidence suggests the next pandemic will likely be a respiratory RNA virus like a coronavirus or influenza (flu) virus, though the threat from other pathogen types remains^22^.

This perspective discusses the current state of preparations for the next pandemic considering key lessons learned from the COVID-19 response and pandemic influenza preparedness, with a particular focus on vaccines. While it is difficult to predict the type and magnitude of the next pandemic pathogen threat, making reasonable assumptions today may enable a more effective response in the future. As with COVID-19, the world may once again rely heavily on non-pharmaceutical interventions (NPIs) amid a disease outbreak, and it may need to deploy stockpiled medical countermeasures—or quickly develop and manufacture new interventions. Observations about past pandemic response and preparedness can guide proactive actions now, which may increase the world’s success in preventing or taming the next pandemic—an ongoing and potentially increasing threat.

## What happened during COVID-19?

**Fig. 1:**
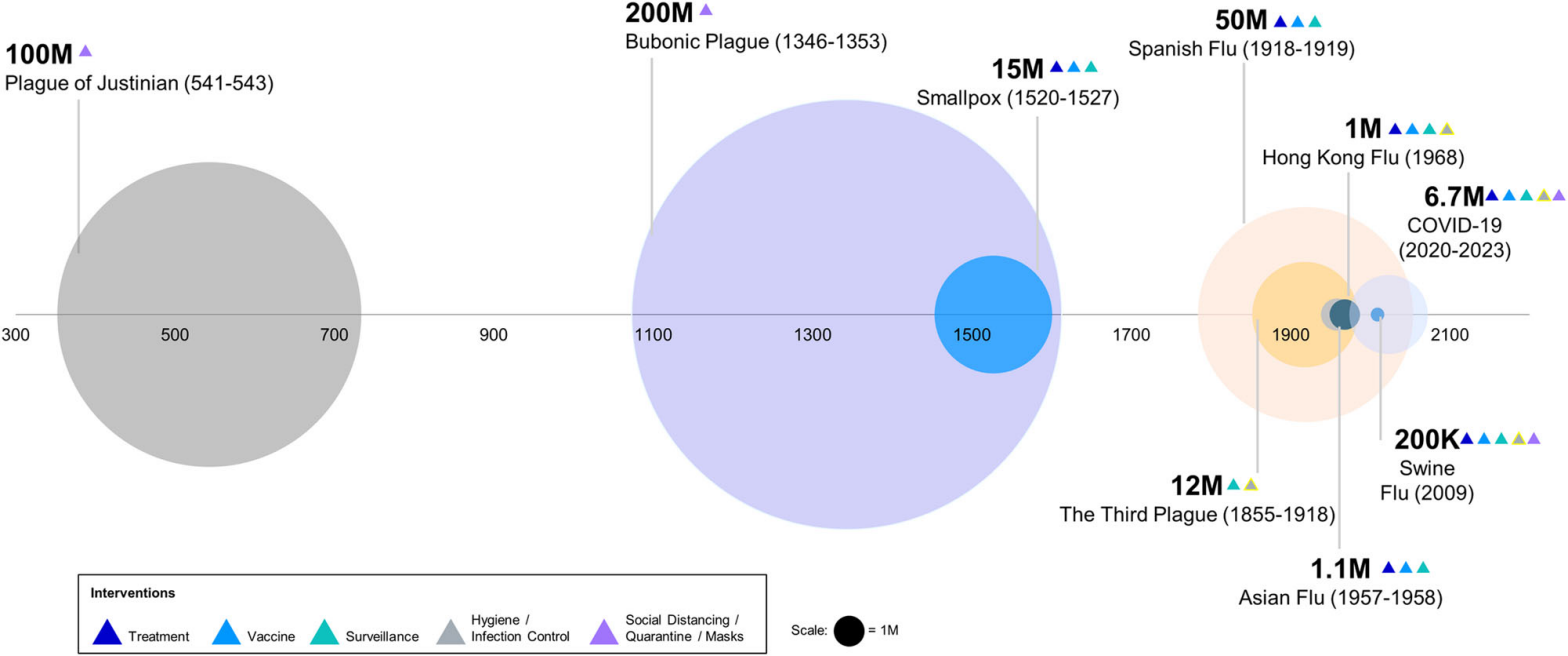
Examples of death tolls from historic epidemics or pandemics. Death tolls of major epidemics or pandemics in the last millennia are signified by circle size. Different interventions (triangles) were used during each pandemic, and advancements in intervention strategies likely reduced death tolls significantly, especially during COVID-19.

Although the health and economic impact of COVID-19 was immense, it could have been far worse had it not been for modern technology (Fig. 1). Historically, pandemics have, at times, reached extraordinary scales; 200 million people died during the bubonic plague between 1346 and 1353, and up to 50 million people died during the 1918 influenza pandemic^23^. While these pathogens differ from SARS-CoV-2 in virulence and transmissibility, they illustrate the potential scale of what can happen without the rapid availability of effective treatments and vaccines.

Similar to some historic epidemics and pandemics, targeted vaccines and therapeutics were not available during the early days of the COVID-19 pandemic^24^. As such, NPIs including lockdowns, social-distancing measures, and mask mandates became priority measures in the early response. NPI implementation and severity differed across the globe but generally included public information campaigns, school and workplace closures, bans on public events, domestic and international travel restrictions, quarantines, and stay-at-home orders^25^. These interventions also played a critical role in reducing disease spread and preserving hospital capacity^25–27^.

In addition to NPIs, the development of new medical countermeasures, including vaccines, was critical to the COVID-19 pandemic response. Countries and multilateral organizations quickly took varying approaches to funding vaccine development, manufacturing, and procurement. Negotiations among manufacturers, governments, and multilaterals ensued even before COVID-19 vaccines were known to be successful. By mid-2020, the US government’s Operation Warp Speed had contracted numerous manufacturers to support research, development, and product procurement across a variety of technologies^28^. Similarly, the Coalition for Epidemic Preparedness Innovations (CEPI) invested in a diverse portfolio of vaccine technologies. The European Commission secured a number of advanced agreements for specific technologies, but it faced challenges to do so quickly given representation across 27 sovereign states with different budgets and risk tolerances^29^. Altogether, governments and multilateral organizations spent billions on medical countermeasures, though early investments represented just a fraction of spending on the COVID-19 pandemic. Indeed, government spending to shore up the US economy exceeded $5 trillion while vaccines and treatments made up about 2% of total spending^30^.

Fortunately, safe, and effective vaccines and treatments were quickly developed and given emergency authorization to begin addressing the burden of COVID-19. The first vaccines were developed in approximately nine months, and to date, more than 13 billion COVID-19 vaccines have been administered^1,31^. The speed of vaccine availability was thanks in-part to flexible strategies used across sectors, including the issuance of emergency approvals by regulatory authorities. For example, the UK’s Medicines and Healthcare products Regulatory Agency (MHRA)—the first stringent regulatory authority to issue an emergency approval of a COVID-19 vaccine—relied on a rolling review process, allowing for the evaluation of clinical data as it became available and thus reducing the time to vaccine roll-out^32^. In fact, this pandemic harbored the fastest response, regulatory emergency authorization, and rollout of a vaccine in history.

Thankfully, the rapid introduction of vaccines and treatments helped to reduce morbidity and mortality of SARS-CoV-2, while helping to alleviate costs. Estimates suggest that vaccinations prevented over 14 million deaths in 185 countries and territories just in the first year of deployment^33^. A recent study conducted in the US suggested that without COVID-19 vaccines, between December 2020 to November 2022, there would have been nearly 120 million more COVID-19 infections, 18.5 million hospitalizations, and 3.2 million deaths^34^. The recent development of antivirals and antibody treatments for use early after symptom onset and for mild to moderate COVID-19 have also begun to further reduce hospitalizations and deaths^34–36^.

Beyond reductions in infections and deaths, research suggests vaccination contributed to major economic benefits. Vaccination may have averted $1.15 trillion in medical costs in the US alone. A local study based in New York City estimated that every $1 invested in COVID-19 vaccine programs yielded a savings of approximately $10 in direct and indirect healthcare costs^37^. The benefits of vaccination continue to progress as ongoing surveillance and speedy manufacturing support the development of updated vaccines for evolving variants of SARS-CoV-2^38^.

While COVID-19 vaccines supported health and economic gains in some places, the pandemic also highlighted the need for strategies to improve rapid, equitable access to vaccines across the world. Higher-income countries had more immediate access to a range of vaccine technologies while lower-income countries faced delays due to additional challenges in financing and operations. The global initiative COVAX (COVID-19 Vaccines Global Access) was created in 2020 to address these issues, striving to provide rapid and equitable access to COVID-19 vaccines and treatments^39^. While admirable and justified, such a goal was also incredibly ambitious. As of the end of 2022, though COVAX had shipped over 1 billion vaccines to 144 countries, only about 22% of people in low-income countries had received at least one COVID-19 vaccine dose^40,41^.

Challenges to vaccine equity in-part underscored risks associated with relying more heavily on single-source suppliers. Despite COVAX’s goal of investments across a range of vaccine technologies, nearly three-quarters of COVAX’s initial supplies were from one supplier in India. When a massive COVID-19 wave hit India in March 2021, vaccine exports were banned, leaving COVAX short 90 million expected doses^42^. Similarly, Australia had limited access to vaccine doses throughout the first half of 2021, having initially counted on the success of a narrow range of vaccine technologies^43^. At-risk investments across multiple technologies and manufacturers aimed to account for significant uncertainties on if, or when, new vaccines would be available.

In sum, COVID-19 represented a novel global challenge, and governments and organizations took various pathways in their responses, including vaccine development and deployment, but extended to a range of other interventions (e.g., use of NPIs or development of new treatments). These experiences can translate into lessons learned and guide preparations for the next pandemic, but first, it is valuable to assess the state of pandemic preparedness before COVID-19.

## The focus on influenza

Until the COVID-19 pandemic caused by the virus SARS-CoV-2, influenza was predicted to be the most likely pathogen to cause the next pandemic due to its high rate of mutation and transmission. In fact, many past pandemics were flu-related; the 1918 influenza pandemic was caused by an H1N1 virus, and smaller-scale influenza pandemics occurred in 1957, 1968, and 2009. Additionally, the World Bank had previously estimated that a moderate or severe flu pandemic could cost $570 billion annually (or 0.7% of global income) highlighting the ongoing threat to global health and economies^44^.

Prior to the COVID-19 pandemic, governments and public health organizations allocated billions of dollars each year for pandemic preparedness and medical countermeasures. Given the potential societal impact of pandemics, preparations for another flu pandemic have been underway for decades, including a focus on stockpiling vaccine products and therapeutics, forging advanced purchase agreements (APAs), research and development toward novel vaccine technologies, and strengthening manufacturing capacity.

Strategic stockpiles are a stopgap in a health emergency, offering critical medical countermeasures when market supplies may be short. The US Biomedical Advanced Research and Development Authority’s (BARDA) Pandemic Influenza Vaccine Stockpile Program stockpiles antivirals and pre-pandemic flu vaccines using egg- and cell-based technologies, as well as adjuvants^45^. BARDA has for example accumulated hundreds of millions of prepandemic H5N1 bulk vaccine doses, with multiple other countries having stockpiled H5N1 vaccines^45^. Stockpiles also extend beyond influenza; Gavi in partnership with UNICEF has routinely stockpiled Ebola vaccines^46^. Another example includes the stockpiling of smallpox vaccines, which were quickly distributed amid a 2022 outbreak of monkeypox^47^. If a pathogen with an outbreak or pandemic potential emerges that closely aligns with a stockpile, it can be quickly distributed to protect the population or provide some level of protection before more tailored interventions are developed if they are necessary. For that reason, stockpiles are one of a set of tools used for pandemic preparedness. One of the limitations of stockpiling is the short shelf life of some products leading to significant financial and material wastage. An additional consideration is that maintaining stockpiles requires stringent inventory management and replenishment. The rise of new SARS-CoV-2 variants every year demonstrates the speed at which pathogen evolution can occur, potentially making stockpiles outdated.

In the case where the next pathogen to cause an outbreak or pandemic is not adequately addressed with stockpiled products, new or updated vaccines will need to be quickly manufactured as was the case with the 2009 H1N1 pandemic. A limiting factor is that some current production processes for influenza vaccines require several time-consuming steps. As per current egg-based manufacturing practices, viral flu vaccines are developed by isolating a new virus strain, growing it in a seed stock, propagating it, and inactivating and purifying it—a process that may take 5–6 months—all before distribution^48,49^. Even still, as the 2009 pandemic showed, the H1N1 influenza virus did not grow well in egg-based medium, the most widely used influenza vaccine technology at the time, contributing to further delays^50,51^.

Given these limitations, research and development has focused on vaccine technology innovation for influenza. A more reliable vaccine technology would lessen the health and economic impact of a pandemic by virtue of an earlier, potentially more effective intervention to slow the spread and health burden of the virus. For example, BARDA supported the development of recombinant influenza vaccines with the goal of reducing the time to vaccine availability in the event of an influenza pandemic, while investments in adjuvants aimed to allow for lower antigen dosing and therefore potentially more supply^52–54^. Recombinant technology and adjuvants were leveraged amid the range of COVID-19 vaccine candidates that arose after the pandemic declaration^55^. In the future, having both existing stockpiles and a fast, reliable—but also flexible—vaccine development process would ensure a more comprehensive level of protection in the event of another pandemic.

Pandemic preparations also extend to the manufacturing and distribution of vaccines. Global manufacturing capacity at any point in time is finite, so it is important to create plans for adequate and thoughtful production and distribution. As previously mentioned, governments and organizations have forged APAs with vaccine manufacturers to prioritize available manufacturing capacity and ensure access once vaccines are available. APAs are agreements to purchase products not yet available, or reserve manufacturing capacity in the case of a disease outbreak, even if the products or capacity are no longer needed by the time they are produced. During the 2009 H1N1 pandemic, APAs were in place to ensure access to products once a pandemic was declared, while further sustaining manufacturing capacity for emergency use before the pandemic phase^56^.

APAs mitigate the risk manufacturers take on when they produce a new vaccine, increasing the likelihood that doses will be available early in a pandemic^56^. Unfortunately, manufacturers may still be limited by slow vaccine production processes. Additionally, APAs may not adequately address issues around vaccine equity. The reservation of supplies by high-income countries with APAs during the 2009 H1N1 pandemic limited initial vaccine orders for lower-income countries and countries without APAs^56^. These limitations underscore the need to reform the design of APAs to prioritize equitable access in a timely manner and invest in vaccine discovery platforms for more rapid vaccine manufacturing.

As an alternative to relying on de novo vaccine development and manufacturing, governments and multilateral organizations have explored the development of “universal” vaccines, though these technologies may be years away. A universal influenza vaccine is one that provides at least partial cross-protection against all influenza strains and variants, even ones that have yet to emerge, thereby potentially preventing severe disease and protecting health systems from being overwhelmed before more strain-specific vaccines become available. Vaccine candidates may target conserved epitopes of influenza viruses that may theoretically be able to provide universal protection against both seasonal and pandemic flu strains. Such a strategy could effectively prevent or mitigate influenza-caused pandemics. To date, no single approach has been successful in late-stage clinical trials. Clinical data for available universal vaccine candidates suggest that these vaccines may not achieve the levels of efficacy needed to control an influenza pandemic^49^. While pursuits toward a universal vaccine continue, a variety of pandemic preparedness and response tactics for influenza, as previously highlighted, must be considered.

Stockpiling, rapid manufacturing through new technologies, and APAs exemplify some of the central approaches to pandemic preparedness today, beyond conducting research and developing pandemic-focused products. These strategies have their individual advantages and limitations, but with robust investment and thoughtful planning, can be improved and leveraged in the next pandemic. Because pandemic preparedness largely focused on influenza before the COVID-19 pandemic, many available interventions were not relevant to the COVID-19 response. Moving forward, broader pathogen-inclusive thinking is warranted when considering relevant preparedness tactics, including stockpiled products and vaccine development and manufacturing technologies. Recognizing that a proactive approach will benefit future populations, it is essential to use lessons learned focused on influenza in the past—as well as the COVID-19 pandemic response—to enhance strategies for pandemic preparedness.

## Lessons from COVID-19 and pandemic influenza

Pandemic preparedness requires comprehensive thinking, from the earliest stages of research to enable effective development of countermeasures, to monitoring pandemic threats, and to coordination of pandemic response efforts in real-time. The time for action is during non-pandemic times, enabling a more effective response in an emergency. While this article highlights lessons specific to pandemic preparedness, many of the recommendations may also apply to local, regional, and global responses to other health emergencies, from smaller-scale disease outbreaks and biosecurity threats to weather-related catastrophes (Table 1).

**Table 1:** Lessons learned to improve pandemic preparedness.

Lesson	Detail
1. Prioritize early-stage R&D and platform technologies	• Invest in basic and translational research, and vaccine platform approaches
	• Improve vaccine platform technologies, including addressing, for example, limitations in storage and distribution
	• Understand vaccine targets and correlates of protection for pandemic pathogens
2. Bolster pandemic pathogen intelligence	• Build on existing genomic surveillance systems to include a range of pandemic pathogens
	• Strengthen local surveillance systems and better connect to global surveillance hubs
	• Use a range of data sources to monitor newly emerging infectious diseases
	• Predict new epidemics or emerging pathogen variants using vast data sources
	• Share disease intelligence to enable the development of new medical countermeasures
3. Optimize and de-risk earlier pandemic interventions	• Optimize the use of non-pharmaceutical interventions before widespread medical countermeasures are available
	• Stockpile essential medicines, hospital supplies, and treatments for surge capacity
	• Diversify response across preparedness tactics, manufacturers, and product technologies to help mitigate single-source risks
4. Sustain and leverage manufacturing capacity	• Sustain “warm base” vaccine manufacturing capacity for use in the next pandemic, including production of vaccines for epidemic or endemic infectious diseases
	• Plan for the expense of new start-up facilities and ongoing investment in infrastructure, operations, and workforce
	• Overcome supply shortages for raw materials to enable rapid vaccine production
	• Consider incorporating surge capacity into manufacturing network planning and operations
	• Promote sustainable procurement policies and health-seeking behaviors to establish routine commercial demand, balanced with appropriate supply capacity
5. Troubleshoot trade, regulatory, and procurement barriers	• Troubleshoot trade and regulatory barriers to the distribution of essential products
	• Harmonize global regulatory pathways to enable accelerated time to market for products
	• Establish more equitable but also sustainable agreements between manufacturers, national governments, and multilateral organizations on how to respond in the event of a future pandemic
	• Establish proactive pandemic funding mechanisms and integrated preparedness plans
6. Leverage the power of partnerships to overcome health system challenges	• Leverage multisectoral partnerships to solve problems of health systems strengthening, supply chains, financing, and consumer engagement
	• Better understand behaviors leading to vaccine hesitancy and leverage insights to develop strategies aimed at increasing uptake

### Lesson #1: Prioritize early-stage R&D and platform technologies

Early-stage research and platform technology approaches enable earlier access to vaccines and treatments. The biggest turning point during the COVID-19 crisis was the deployment of vaccines; and vaccines using novel platform technologies, like mRNA and adenovirus platforms, were among the fastest to be developed and authorized. Technology platforms are frameworks that allow the development of new vaccines without customizing the process, allowing for rapid production of multiple vaccines from a single system. The speed and flexibility of vaccine platforms contributed to reducing overall morbidity and mortality from COVID-19, which eventually lowered reliance on NPIs to slow disease spread^35,36^. The earlier a vaccine is made available in a pandemic, likely the more favorable the outcome^33^.

Thus, world leaders have set ambitious goals to respond more swiftly to the next pandemic. The US set goals to design, test, and review a new vaccine just 100 days after a pandemic declaration and to produce enough vaccines for the US and the world in 130 and 200 days, respectively^57^. Similarly, both CEPI and the G7 have initiatives that aim for new vaccines to be ready for authorization within 100 days after recognition of a pandemic pathogen^58,59^. Such speeds will require streamlining existing processes, like increasing collaboration and information sharing between government and industry and faster approval processes.

Crucially, the mRNA vaccines developed to combat SARS-CoV-2 were not an overnight success. Development of the COVID-19 mRNA vaccines was enabled by decades of research following the initial production of synthetic mRNA in the 1980s^60,61^. Equally important, advancements in carrier lipid nanoparticles enabled the delivery of mRNA to cells^62^. mRNA’s use as a therapeutic has been examined since the 1990s but was finally demonstrated at a global scale during the COVID-19 pandemic^61^. Importantly, research conducted by the US National Institute of Allergy and Infectious Diseases (NIAID) on both severe acute respiratory syndrome (SARS) and Middle East respiratory syndrome (MERS) revealed the spike protein as a target for vaccine development, allowing for rapid production of mRNA vaccines against SARS-CoV-2^49,58^. Further research discovered that the 2 P stabilization of the spike protein was a modification that helps to stabilize the S protein in its prefusion form, which is a target for the immune response and therefore crucial for vaccine efficacy^63^. The fundamental role of basic research cannot be overstated. The success of the COVID-19 mRNA vaccines relied on years of progress in basic and translational research on influenza and previous coronaviruses^60^. Therefore, continuing to invest in basic research, as well as flexible vaccine development platforms, could help to speed response to the next pandemic^58,64^.

Basic and translational research must continue following COVID-19 because the next pandemic pathogen may be even harder to target than SARS-CoV-2^60^. Ongoing research should be informed by surveillance systems that track pathogens with the potential to cause an outbreak or pandemic. Understanding vaccine targets and correlates of protection of these pathogens and generating data may provide the solid foundation of science needed for rapid vaccine development. This process requires continuous funding, yet typically, there are valleys in funding that follow high peaks during a disease outbreak or pandemic. This was highlighted by the lack of sustained, continuous investment in vaccine research following the SARS outbreak in the early 2000s, which affected the development of new vaccine technologies^49^. Commitments to maintain funding for vaccine research from both public and private funds—and an acceptance of funding research with a higher risk of failure, given difficult-to-target pathogens—may ensure rapid development of a vaccine when a new pathogen emerges.

Some initiatives are already committed to funding research with the aim of advancing our understanding of various virus families and developing effective vaccines. NIAID, for instance, focuses on studying potential pandemic-causing viruses, and CEPI is aiming to develop a comprehensive library of prototype vaccines against a range of viral pathogen families^65–67^. While these efforts could be complicated and slow given the range of pathogens of outbreak and pandemic potential, early-stage R&D initiatives such as these may provide enough learnings to jump-start future pandemic responses.

Further, agile vaccine technology will be critical for the response to any future pandemic due to the unpredictability of emerging pathogens. Existing mRNA vaccine platforms are highly suitable for a rapid response to an emerging pathogen given their proven manufacturing agility and scale, as demonstrated during the COVID-19 pandemic^68^. Typically, manufacturing can commence shortly after the antigen genetic sequence has been ascertained. This could result in both timely and effective responses to emerging threats from influenza, coronaviruses, or other pathogens with pandemic potential. Alongside an adaptable vaccine platform, research should also focus on addressing limitations in vaccine storage and distribution. For current mRNA vaccines, the requirement to keep doses frozen is a significant barrier to global distribution^69^. There exist goals to develop a more “ideal” vaccine, one which has a longer shelf life, extended durability, minimal dosing schedule, and wider breadth of coverage^70^.

Currently, efforts are also underway to apply mRNA technology to influenza following the proven success against SARS-CoV-2^71–73^. But this technology may also hold promise for other endemic pathogens for which vaccines have been difficult to develop or pathogens with significant outbreak or pandemic potential^74^. Efforts are needed to steer research funding toward a better understanding of pandemic pathogens and vaccine targets; projects such as the WHO’s recently launched process to update their list of pathogens with pandemic potential may begin to accomplish this^75^.

The benefit of vaccine technologies such as mRNA relies on their “plug and play” possibilities to allow for a flexible response in the next pandemic, enabled by research identifying the most effective vaccine targets for a range of pandemic pathogens. Unlocking the potential of platform technologies will require collaboration across governments, multilaterals, academia, and industry to prioritize it. In the future, mRNA vaccine technology will be an important tool among a suite of options to respond to pandemics, one that has already been proven as an effective platform on a global scale.

### Lesson #2: Bolster pandemic pathogen intelligence

Detecting novel pathogens as they arise allows for the earliest possible response, so surveillance systems should be expanded and more extensively leveraged to better detect and respond to infectious disease outbreaks in real time. Sentinel surveillance systems for global influenza—e.g., the Global Influenza Surveillance and Response System (GISRS)—were leveraged during the COVID-19 pandemic and could continue to play an important role for SARS-CoV-2 and future pathogens. In partnership with WHO, GISRS was systematically expanded to include RSV in 2015 and SARS-CoV-2 in 2020, and vitally acting as early testing centers for SARS-CoV-2^76^. Strengthening systems like GISRS to include even more pathogens of outbreak or pandemic potential can improve future surveillance efforts. In parallel, there is a need to expand the number of surveillance sites globally; more than 70 countries still lack WHO-designated influenza surveillance centers, let alone broader systems^77^.

Excluding systems for West Nile virus and other arboviruses, no formal system exists to actively monitor a broad range of priority emerging and re-emerging infectious diseases, both in animals and humans^78,79^. For respiratory diseases, using existing influenza surveillance systems to monitor outliers of influenza-like illness (ILI) more extensively, which may encompass a range of pathogens, could also lead to earlier outbreak detection. One study speculated that if such a robust surveillance system were in use, the spread of COVID-19 could have been detected more than 13 weeks before the first reported infection peaks^80^. Earlier detection of SARS-CoV-2 could have led to an earlier response, potentially limiting its health and economic impact.

An expanded global surveillance system would also require investment in laboratory infrastructure, diagnostic capabilities, and workforce development at a local-, national-, and international level. Projects such as the Seattle Flu Study and the US Agency for International Development’s PREDICT may provide a roadmap. The Seattle Flu Study, launched in 2018 by the Brotman Baty Institute, University of Washington School of Medicine, Seattle Children’s Hospital, and the Fred Hutchinson Cancer Research Center, is a city-wide platform for the surveillance of respiratory pathogens, as well as pilot interventions^81^. This platform was used to identify the first documented U.S. case of COVID-19 community transmission in February 2020. PREDICT, which operated across more than 30 countries for a decade, worked from the ground up to strengthen surveillance for both known and newly discovered viral threats. Given lessons from COVID-19, it may also be time to experiment with new models of building surveillance systems at the local level^82^. Community-based surveillance, particularly in low- and middle-income countries, integrated with national and global surveillance hubs, such as WHO’s newly launched Hub for Pandemic and Epidemic Intelligence, could help drive earlier detection of emerging infectious diseases^83,84^.

With information on pathogens coming from surveillance systems, major public health authorities have evolved strategies to constantly evaluate pandemic risk. The CDC’s Influenza Risk Assessment Tool (IRAT) and WHO’s Tool for Influenza Pandemic Risk Assessment (TIPRA) evaluate the risk of viruses not currently circulating in humans and help to prioritize investments in pandemic preparedness^85,86^. For example, changes in the viral properties of a particular flu strain may signal the need to assess this strain for pandemic potential^86^. These tools may guide research and surveillance, while also serving as a forum to share information between scientists, public health authorities, and other stakeholders. They may also facilitate the development of pre-pandemic vaccines; this happened following the emergence of the pandemic flu strain H7N9 in 2013^48^. As in this example, disease intelligence must be translated into action.

Furthermore, to effectively respond to newly detected disease outbreaks, sharing pathogen data is essential. Originally established in 2011, the WHO’s Pandemic Influenza Preparedness (PIP) Framework allows for pathogen samples to be shared with companies to support vaccine development. In exchange, manufacturers agree to approaches that increase access to pandemic vaccines, thereby increasing equity in the event of a pandemic^87^. Efforts like these lay the groundwork for data sharing in a future pandemic, but must not require additional negotiation in the event of a crisis, leading to delays in the development of medical countermeasures. For example, varied national interpretation of the Nagoya Protocol—a supplemental agreement to the Convention on Biological Diversity (CBD) that came into effect in 2014—has led to delays in sharing virus samples and subsequent manufacturing for seasonal influenza vaccines^88^. During COVID-19, China’s sequencing and sharing of the SARS-CoV-2 genome just days after identifying it was pivotal to successful vaccine development^89^. Continuous genetic sequencing of the circulating virus then allowed the detection of variants as they emerged^90^. Sequencing combined with surveillance may uncover the next SARS-CoV-2 variant or novel pathogen before it escalates, as long as frameworks are in place to rapidly disperse this information to the world.

On top of detection and assessment, an optimal disease intelligence system would seek to predict the next pathogen with pandemic potential. Vast amounts of existing data can be used to inform decision-making on pandemic policy and response through, for example, predictive modeling^91^. Other efforts are underway to use artificial intelligence to predict the next pathogen spillover event^92^. There is an opportunity to test new approaches to predict emerging pathogens using a range of data sources while ensuring surveillance systems focus on threats of respiratory pathogens^93^. Above all, sharing disease intelligence and data—quickly, in the event of a disease outbreak—can speed response to the next pandemic.

Lastly, it is pertinent to assess how to ensure prompt reactions when surveillance systems ring the alarm. In January 2020, the WHO sounded the pandemic alarm for COVID-19, yet few countries responded immediately^94^. Many of the calls to action by WHO were ignored, such as suggestions to quickly begin testing and social distancing^95,96^. These delays occurred for many reasons and varied from country to country. Some governments exercised caution to not disrupt their people’s livelihood and economy, and others lacked an understanding of the pandemic signifier itself^94^.

One suggested approach to kick-starting earlier vaccine development is to have a gradient of warnings that separate dangerous pandemics from more manageable outbreaks. This system, akin to the early warning systems used in healthcare and weather-related scenarios^97–100^, could be employed for pandemic preparedness. For instance, in healthcare settings, an artificial intelligence platform could help prioritize patients based on their medical needs, effectively managing resources during triage situations^100^. Similarly, a gradient-based warning system for pandemics could initiate appropriate responses at different levels of threat, with each level tied to specific actions. An early warning or Level 1 may involve increased surveillance and information sharing, while higher levels could trigger more drastic measures like regional shutdowns or global travel restrictions. Low-grade alarms may also result in more active information sharing by governments since an innocuous signal would diminish fears of causing panic and disrupting economies^94^. However, the success of such a system hinges on complete adherence to the rules it prescribes. An incomplete application could potentially lead to inefficiencies or confusion, but despite this, even partial application of these systems could prompt earlier responses and slow down the spread of a pandemic.

Undeniably, the evolution of disease surveillance and intelligence systems is not simple. It requires major investment and coordination across global-, national-, and local levels. The inherent complexity requires cooperation across borders, and strong leadership from global health actors given the need to quickly act and share intelligence globally. Strengthening leadership and funding at the global level may help with coordination, but countries must also commit to sustained cooperation in the short- and long-term^15^.

### Lesson #3: Optimize and de-risk earlier pandemic interventions

NPIs saved lives but are not without limitations and consequences. Persistent long-term reliance on NPIs can be challenging because people grow tired and apathetic toward them^101^. Another limitation is that there may not be, or necessarily should be, a universal strategy for NPIs. Differences in NPI timing, intensity, and adherence showed varying levels of success, demonstrating the importance of geographically specific and informed NPI policies^102^. Research from countries that imposed lockdowns showed that while NPIs were very effective at controlling spread, they resulted in significant economic, social, and health costs^26,102,103^. Some consequences were clearly visible, like increasing unemployment rates from business and school closures and the spike in non-COVID-19 deaths due to the unavailability or avoidance of medical care^7,26^. Others, like the effect of social distancing on the mental health of children and adolescents, continue to be difficult to measure.

Beyond NPIs, early action should aim to leverage all available interventions as soon as possible in pandemic response, which may require pre-planning strategies and stockpiling a broad range of essential supplies. While leaders were encouraging social distancing early during the COVID-19 pandemic, US hospitals were already reporting shortages in basic supplies and essential medicines^104^. Shortages were amplified by supply chain bottlenecks, which limited access to many basic supplies, including personal protective equipment (PPE) for frontline hospital staff^104^. The shortage of facilities and pharmaceutical glass, especially Type I glass vials used for vaccines, also strained fill-finish capacity^105^. The existing fill-finish capacity shortages were further intensified by the pandemic due to a shift from vials to syringes and cartridges, increasing the demand for syringe capacity^106^. In response, companies reprioritized their manufacturing networks to ensure adequate production of supplies such as sterile injectables and PPE. In preparation for the next pandemic, several improvements can be made to ensure adequate supplies of essential products for healthcare systems. Governments and health systems may pre-plan access to essential medicines, hospital supplies, and treatments in anticipation of growing needs. They may also discourage the use of medical supplies, like PPE, in nonmedical settings and redirect those supplies to the most overburdened areas^104^.

Beyond targeted medical countermeasures, NPIs and emergency supplies represent the need to think broadly about what is needed to respond most effectively in the earliest days of the next outbreak or pandemic. NPIs will continue to be an early mainstay of pandemic response, and research has suggested interventions such as physical distancing can be cost-effective^107^. Additional research may inform the most timely and locally acceptable ways to roll out NPIs in the future. Furthermore, scenario planning may help pre-plan NPI strategies, as well as contents of strategic stockpiles moving forward, employing lessons learned from COVID-19.

These actions along with NPIs may significantly curb the spread of a pandemic virus, but ultimately, the earlier availability of medical countermeasures like vaccines and treatments is needed. In the time before tailor-made solutions are available, governments may utilize all available “off the shelf” solutions, including stockpiled products, to blunt the impact of disease outbreaks. WHO, for example, recommends the stockpiling of influenza antiviral therapeutics to reduce mortality in a pandemic^87^.

The response to COVID-19 required a rapid end-to-end response, which may again be the case in the next outbreak or pandemic. COVID-19 demonstrated the importance of diverse interventions—including NPIs and medical countermeasures—across a range of preparedness tactics, manufacturers, and product technologies to help mitigate risks. While it is impossible to predict the efficacy of any single intervention in the next pandemic, planning to employ a range of responses can guard against the risk any single intervention will not work or be available in an emergency.

### Lesson #4: Sustain and leverage manufacturing capacity

New vaccines rely on manufacturers to make them. Adequate vaccine manufacturing capacity—scalable and aimed at rapid deployment—is vital. At the start of the COVID-19 pandemic, early surge capacity was inadequate to meet demand, and prior research had already predicted this would be the case^108^. But since 2020, manufacturers have scaled up to unprecedented capacity. The International Federation of Pharmaceutical Manufacturers and Associations (IFPMA) estimated that worldwide vaccine manufacturing capacity would reach 12.5 billion by the end of 2021^109^. More recent data suggests this number will reach 20 billion by the end of 2022^110^.

Momentum is growing to create additional end-to-end vaccine manufacturing capacity in low- and middle-income countries^111^. Local manufacturing seeks to overcome barriers around unequal vaccine distribution and trade restrictions experienced during the COVID-19 pandemic. The largest barriers for new manufacturers appear to be cost and demand. Manufacturers building new facilities may need to price vaccines higher than global competitors to cover high start-up costs, and institutional buyers may need to be prepared to absorb the premium despite limited financing^112^. Furthermore, the sustainability of those facilities is directly tied to demand for vaccine production—if demand is limited, local production will be threatened. This has been the case for manufacturing COVID-19 vaccines in South Africa. Despite technology transfer to manufacturers for locally produced vaccines, health authorities reported limited purchasing by African countries given the availability of free doses elsewhere^113^.

One potential solution could be found with international organizations helping to guarantee demand for vaccines to support investments into new manufacturing. For example, the Pneumococcal Advance Market Commitment (AMC) has helped ensure access to pneumococcal vaccines in developing countries by guaranteeing a market for vaccines before development^114^. Another potential solution is to produce a range of routine immunizations beyond pandemic vaccines^112^. There are several vaccine markets that currently have high demand but a low number of suppliers, such as measles, rubella, cholera, and malaria^112^. The suitability of mRNA technology for vaccine development for these and many other pathogens remains unknown at this time. However, if new manufacturers produce vaccines that are needed on a routine basis worldwide, they may sustain their manufacturing capacities despite the potential for higher costs early on. But new manufacturers also need to ensure that there are buyers for these vaccines. Therefore, alone, this strategy is not enough, but with multilateral organizations like Gavi prepared to purchase from these new facilities at risk, along with other countries in the region, new manufacturers would be set up for greater success. International efforts to strengthen routine healthcare systems, including last-mile delivery of health products, and encouraging health-seeking behaviors, could help further build the demand needed to sustain local facilities.

Certain countries may also need to further develop and standardize regulatory systems surrounding medical products^115^. Additional regulatory expertize may be needed because many countries lack robust regulatory agencies, which may slow or limit the development of local manufacturing facilities and their approval or the acceptance of products from other countries. Multilateral organizations and governments are helping to address these challenges with the goal of ensuring biosecurity for lower-income countries before the next pandemic, though ensuring the sustainability of new manufacturing facilities is crucial.

Efforts to further localize manufacturing in low- and middle-income countries may take years to build up, so while these efforts continue to evolve, it is beneficial to sustain and use the capacity currently available. Existing facilities can be leveraged as a reliable source of production capacity in a future pandemic if it is sustained over time. Sustained capacity will require regular investments in infrastructure and operations and a trained workforce. Additionally, there will be a need for steady supplies of raw materials to support vaccine manufacturing in the event of a pandemic. Raw materials for mRNA vaccine production have been costly and scarce given the novelty of the platform. Harnessing the major benefits of an mRNA vaccine platform would therefore require careful management of raw material suppliers, at least in the near term. Establishing raw material stockpiles may prove useful in absorbing the initial need during a pandemic. With basic components in place, existing manufacturing facilities can be “warm”, primed, and ready to respond to a future pandemic. Global manufacturers have a role to play in ensuring capacity is allocated fairly, above the interests of any one country. Ultimately, international collaboration will be the key to ensuring everyone, everywhere, has access to life-saving vaccines.

### Lesson #5: Troubleshoot trade, regulatory, and procurement barriers

Viruses do not have a nationality, yet vaccine nationalism—governments reserving vaccines for their own populations, leaving limited access for the rest of the world—was a pervasive problem during COVID-19. Vaccine nationalism and a lack of regulatory harmonization slowed the movement of vaccines, health products, and essential supplies across borders. Early decision-making in the COVID-19 pandemic was influenced by national interests, underscored by the uncertainty of a new outbreak. The resulting inequality of vaccine access posed a danger to all individuals as the virus spread across borders. Despite this, many high-income countries quickly developed procurement agreements for vaccines to cover their own populations. Meanwhile, COVAX, as a new organization representing many low- and lower-middle-income countries, faced challenges to begin operations^116^. Manufacturers honored commitments in the order of when contracts were forged, which ultimately meant many high-income countries had earlier access to vaccines than lower-income countries.

Trade and regulatory barriers further slowed the manufacture and distribution of essential products^117^. During the COVID-19 pandemic, countries repeatedly prioritized their own populations even though pandemics are, by definition, a global concern. In response to COVID-19, many countries imposed export restrictions on essential products. For example, in March 2021, Italy refused shipment of 250,000 vaccine doses to Australia^118^. When India halted vaccine exports, as previously discussed, it heavily affected the ability of COVAX to serve low- and lower-middle-income countries^119^. The US and EU also restricted the export of raw materials used for vaccine production^119^. These issues could potentially be overcome with pre-established agreements for the distribution of vaccines, health products, and raw materials coupled with efforts to ensure adequate manufacturing capacity is kept “warm.” These incidences reiterate a need to not only establish proactive mechanisms that foster a more robust response in the future but to do so in a fair, diplomatic, and equitable manner.

Preplanning supply agreements and determining in advance how manufacturing capacity is used in the event of a pandemic can enhance the safety of all countries before an emergency occurs. Establishing agreements (e.g., APAs) among national governments, multilateral organizations, and manufacturers with diverse vaccine technology platforms on how to respond in the event of a future pandemic could support the rapid delivery of new vaccines and treatments and help manufacturers reduce uncertainty around supply and demand. Furthermore, agreements should aim to achieve greater health equity by covering countries across income ranges. Tiered pricing of agreements and early considerations for vaccine donations, along with efforts to reserve capacity specifically for low- and lower-middle-income countries, are tools that may provide equitable access to a suite of necessary health interventions in the early days of a pandemic. This would ensure early access to pandemic vaccines and therapeutics in parallel across all countries.

To support preparedness, governments and multilateral organizations should establish sustainable and proactive global funding mechanisms, with clear policies and governance. Efforts are already underway to develop long-term funding mechanisms for low- and middle-income countries to invest in prevention, preparedness, and response. These tailor-made mechanisms may help ease and hasten the initial pandemic response and overcome bottlenecks to financing, as experienced for example by COVAX^120^.

Importantly, global financing and preparedness platforms can also further incentivize local investments and planning, including the development of integrated preparedness plans at the national level^121^. Experts recommend these country-level preparedness plans should include a focus on allocating emergency financing toward preparedness, in addition to surveillance and monitoring and connection to robust commodity supply chains, including therapeutics and vaccines, among other areas^122^. By prioritizing health equity goals and creating a mechanism through which high-income countries and multilateral organizations can collaborate on pandemic preparedness, these funding platforms may also further avoid vaccine nationalism felt during the COVID-19 pandemic.

Lastly, greater governance and regulatory cooperation, and harmonization would speed up access to new health technologies during a pandemic. Even with initiatives already in place, like the International Coalition of Medicines Regulatory Authorities (ICMRA), COVID-19 caused trade restrictions that led to imbalances in supply and demand across the globe. Countries, multilateral organizations, and manufacturers should commit to building harmonized frameworks across borders that prevent these issues in the future and consider flexibilities including rolling reviews and consensus on the use of real-world data, where appropriate. In addition, pre-existing regulatory approvals for components of vaccine platforms, such as the components of mRNA platforms, may mean only targeted, supplementary regulatory submissions would be necessary in the event of an emergency. The ideal future situation includes regulatory submissions whereby manufacturers could effectively disseminate a robust, standardized data package to support regulatory approvals across multiple markets at the same time. Moreover, thoughtful indemnification and liability schemes could be developed in advance to enable innovation in an emergency. Streamlined, widely adopted efforts like these would quicken global response.

### Lesson #6: Leverage the power of partnerships to overcome health systems challenges

Strengthening health systems may be another lever through which the world may better prepare for the next pandemic. Essential healthcare infrastructure, from service delivery, health workforce, supply chain, financing, and consumer engagement provides the basis through which emergency response may operate. The response to COVID-19 showed the power of international, cross-sector collaboration, as well as partnership across governments, multilateral organizations, and the private sector to solve health systems challenges.

While partnerships across sectors are not new, the COVID-19 pandemic should provide more momentum to achieve even bigger goals, including new ways of working. For example, Pfizer and BioNTech partnered with Zipline to provide not only financial support but also technical assistance to enable the world’s first drone delivery of COVID-19 vaccines requiring ultra-cold chain in Ghana^123^. Pfizer also collaborated with the Global Environment and Technology Foundation to collaborate with Project Last Mile. The partnership focused on aligning the supply chain expertise and technical capabilities of Coca-Cola, a company whose supply chain is characterized as having one of the widest reaches in the world, with technical expertise from Pfizer on vaccine handling, storage, and administration to improve the availability of vaccines in developing countries. These and other examples show the biopharmaceutical industry can play a role in health systems strengthening beyond serving as only a manufacturer and transactional supplier of medicines and vaccines^124^.

One area ripe for cross-sector collaboration is tackling vaccine hesitancy. Given the wider global availability of COVID-19 vaccines, the primary roadblocks to vaccine uptake continue to include vaccine hesitancy^122^. In the United States, almost three years into the pandemic, nearly one-third of people are not fully vaccinated for COVID-19—and research shows that hesitancy is prevalent worldwide^125,126^. The challenge of vaccine hesitancy could continue to limit COVID-19 vaccine primary and booster uptake, but also the uptake of both routine immunizations and future pandemic vaccines.

As decision-makers for their own health and that of their children, adult consumers are a primary audience for efforts to increase vaccine uptake. Making routine adult vaccination a mainstay of every clinical visit, beginning with primary care as is done for pediatric vaccination, could potentially help address hesitancy. Public health leaders, governments, and manufacturers may integrate lessons learned from other therapeutic areas as well; for example, making HIV testing more routine in primary care visits has been shown to increase diagnosis and linkage to care. A better understanding of behaviors leading to vaccine hesitancy—and designing solutions to address them—could increase the success of vaccination campaigns in a future pandemic. Multiple stakeholders may all have a role to play, thus collaboration remains key^127^.

## Conclusion

**Fig. 2:**
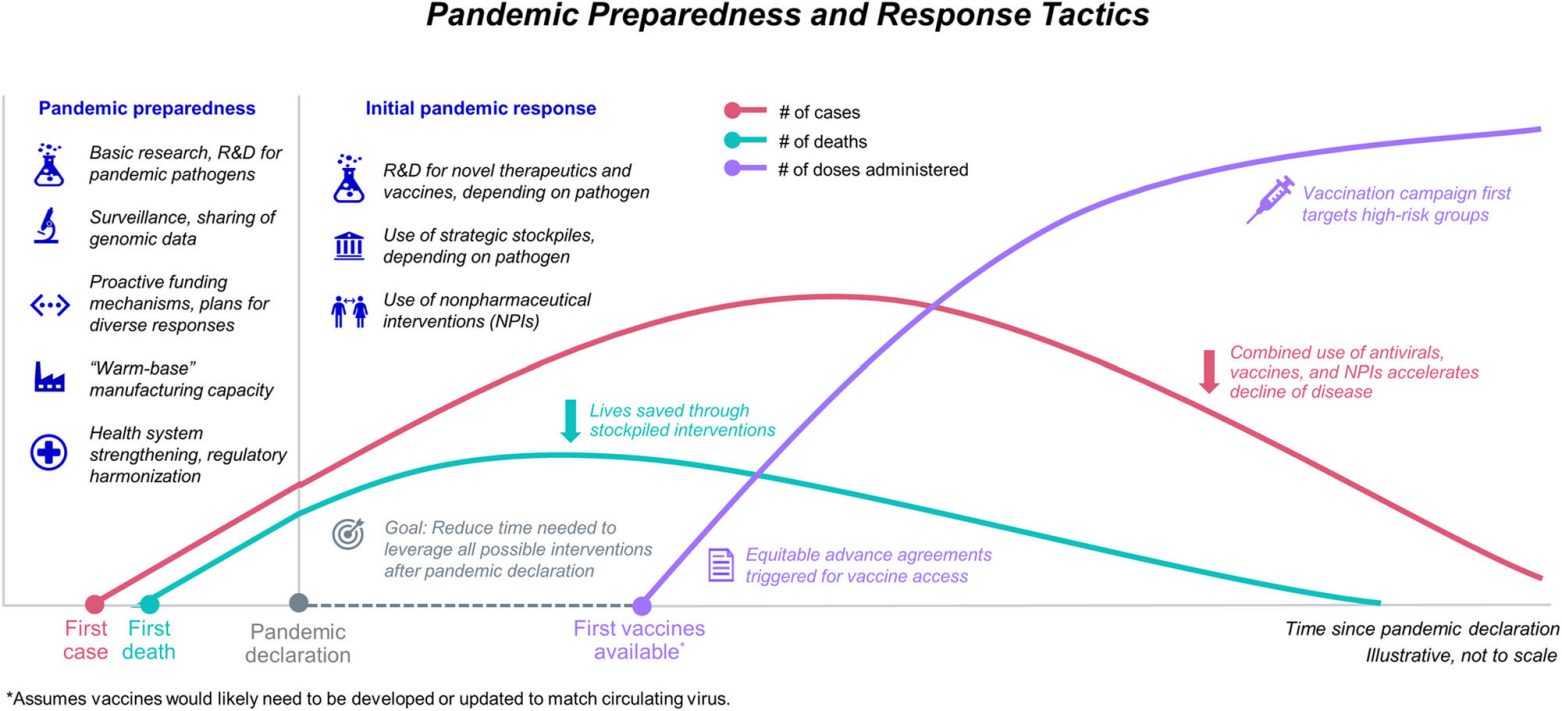
Applying lessons from COVID-19 to reimagine the future. The number of cases (pink line), number of deaths (teal line), and number of vaccine doses administered (purple line) are all influenced by pandemic preparedness and response tactics. Decreasing the delay between responses and the point of pandemic declaration would lower the number of cases and deaths earlier in a pandemic. Vaccination is a key intervention for this result.

COVID-19 had an historic impact that will be felt for years to come, but the risk of another pandemic is ever-present even as the memory of COVID-19 fades. The swift response included an unprecedented collaboration and movement of resources to save lives, develop new systems, and forge partnerships (Fig. 2). These actions were important in alleviating the health and economic burdens of COVID-19, but it is also clear that improvements can be made to ensure readiness for the next pandemic—and these efforts may also aid broader response to other widespread health emergencies.

Advancements and refinements can be implemented throughout every point of the pandemic response, from sentinel surveillance systems to global vaccine distribution. Investing in innovative vaccine technologies and platforms, sustaining manufacturing capacity, facilitating local manufacturing where necessary, and forming regulatory agreements proactively, will be some of the first steps.

There should also be a focus on maintaining global momentum around preparedness and avoiding complacency. Despite the costs of responding to public health emergencies estimated in the billions and trillions of dollars, average spending for public health preparedness in higher-income countries has stayed relatively flat or decreased over time^128^. Bolstering proactive and dedicated financing for preparedness and working to pre-establish cross-border and cross-sector agreements may also help to achieve greater health equity in the next pandemic response.

Interlaced amongst all potential initiatives is an increased need for cooperation in the global community. Each player is part of a global ecosystem, and every type of organization—including the private sector—has a role to play. Protection against a global threat like a pandemic pathogen requires a united front that is built upon collaboration. The risk of another pandemic is ever-present and real, but there can be countless lives saved tomorrow if everyone comes together today.

## Data Availability

No datasets were generated or analyzed for this manuscript.
